# The Essential Oil of *Hyptis crenata* Inhibits the Increase in Secretion of Inflammatory Mediators

**DOI:** 10.3390/plants11223048

**Published:** 2022-11-11

**Authors:** Rutyleia Alves-Soares, Hermógenes David de Oliveira, Dyély de Carvalho Oliveira Campos, Yuri de Abreu Gomes-Vasconcelos, Francisco Walber Ferreira-da-Silva, Kerly Shamyra Silva-Alves, Lianna Noronha Coelho-de-Souza, Lúcio Ricardo Leite Diniz, José Henrique Leal-Cardoso, Andrelina Noronha Coelho-de-Souza

**Affiliations:** 1 Postgraduate Program in Physiological Sciences, Superior Institute of Biomedical Sciences (ISCB), Campus do Itaperi, State University of Ceara (UECE), Av. Silas Munguba 1700, Fortaleza CEP 60.714.903, Ceara, Brazil; 2 Postgraduate Program in Biochemistry, Department of Biochemistry and Molecular Biology, Campus do Pici, Federal University of Ceara (UFC), Av. Humberto Monte, s/n Bloco 907, Fortaleza CEP 60440-990, Ceara, Brazil; 3 Graduate Program of Medicine, Center for Health Sciences (CCS), Fortaleza University (UNIFOR), Av. Washington Soares 1321, Edson Queiroz, Fortaleza CEP 60811-905, Ceara, Brazil; 4 Researcher at the National Institute of the Semiarid Region (INSA), Av. Francisco Lopes de Almeida, s/n Serrotão, Campina Grande CEP 58434-70, Paraiba, Brazil

**Keywords:** medicinal plants, essential oil, *Hyptis crenata*, anti-inflammatory activity, mechanism of action, cytokines

## Abstract

Background: *Hyptis crenata* is a plant of great ethnopharmacological importance widely distributed in South American countries. In Northeast Brazil, teas or infusions of its aerial parts are used in folk medicine to treat several acute and chronic inflammatory diseases. In a previous work we have demonstrated that the essential oil of *H. crenata* (EOHc) has an antiedematogenic effect. The aim of this work was to evaluate the effect of EOHc on cytokines secretion and cellular infiltration. Methods: Peritonitis and paw edema models induced by carrageenan were used to determine leucocyte count, myeloperoxidase (MPO) activity, nitrite, and cytokines secretion. Results: EOHc (10–300 mg/kg) significantly inhibited leucocyte migration and reduced the neutrophil count (control: 1.46 × 10^3^ ± 0.031 × 10^3^/mL) of the total leucocytes population in extracellular exudate (control: 2.14 × 10^3^ ± 0.149 × 10^3^/mL) by 15.00%, 43.29%, 65.52%, and 72.83% for the doses of 10, 30, 100, and 300 mg/kg EOHc, respectively (EC_50_: 24.15 mg/kg). EOHc (100 mg/kg) inhibited the increase in myeloperoxidase activity and completely blocked the increase in nitrite concentration induced by carrageenan. EOHc markedly reduced the pro-inflammatory cytokines (IL-6, MCP-1, IFN-γ, TNF-α, and IL-12p70) and increased IL-10, an anti-inflammatory cytokine (compared to control group, *p* < 0.05). Conclusions: This study demonstrates that EOHc has a long-lasting anti-inflammatory effect mediated through interference on MPO activity, and nitrite, and cytokines secretion. This effect, coupled with low EOHc toxicity, as far as results obtained in mice could be translated to humans, suggests that EOHc has great potentiality as a therapeutic agent.

## 1. Introduction

*Hyptis crenata* (genus Hyptis) — known as “salva-do-Marajó”, “hortelã-do-campo”, and “hortelãzinha” [1] — is a plant that occurs in several South American and Asian countries. In Brazil, it is present in several states, usually on wet soils, near banks of rivers and streams, like those in the North and Northeast [2] in the Amazon rainforest, Atlantic rainforest, Pantanal region, and Cerrado regions [3,4]. *H. crenata* bears a particularly rich distribution in the Amazon River estuary, the Marajó archipelago, and the states of Pará and Amapá [5]. It has also been reported in other regions and countries in South America, such as Bolivia [6]. This plant is of great ethnopharmacological importance. In Northeast Brazil, teas or infusions of its aerial parts are widely used in folk medicine to stimulate appetite and to treat several acute and chronic inflammatory diseases like swelling, gastrointestinal disorders, and arthritis [2,7,8,9,10].

Our research group has shown that the essential oil of *H. crenata* (EOHc) bears low toxicity [11] and both gastroprotective [2] and hepatoprotective activities [9]. Recently we have shown that the essential oil of *H. crenata* presents long-lasting antiedematogenic effect in models of edema induced by carrageenan or dextran in mice, which suggested anti-inflammatory activity as its mechanism of action [10].

This study, however, did not included important aspects of the inflammatory mechanism, such as cytokines release and cellular infiltration. In this work, therefore, we investigated the effect of EOHc on cytokines secretion and cellular infiltration in order to further elucidate the role of these important factors on its mechanism of action.

## 2. Results

The EOHc here employed is the same sample used in Coelho-de-Souza [10]. The chemical analysis of this sample revealed that the major constituents were camphor (33.62%), 1.8-cineole (eucalyptol) (19.76%), and α-pinene (15.24%) [10].

### 2.1. Effect of EOHc on Leucocyte Migration 

**Figure 1:**
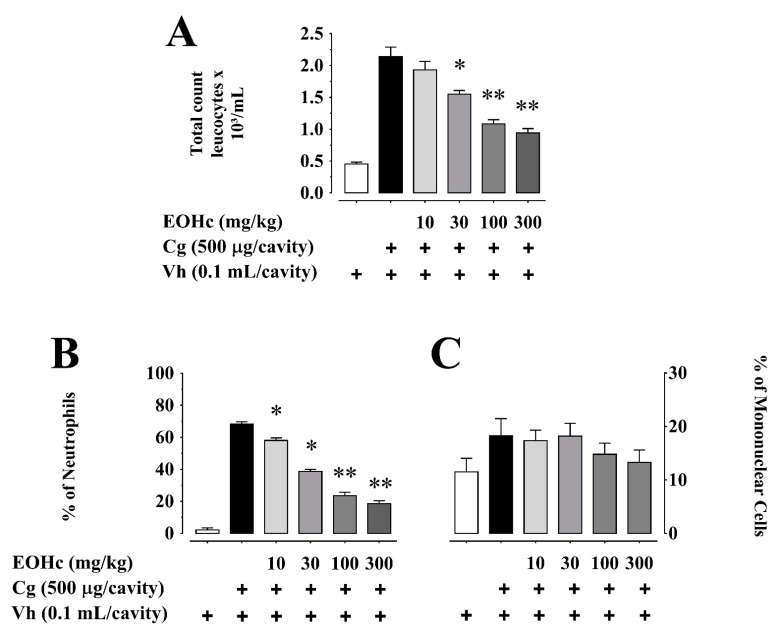
Inhibitory effects of essential oil of *Hyptis crenata* (EOHc) on peritonitis induced by carrageenan. EOHc (10–300 mg/kg) or the vehicle (0.1% Tween 80) was administered, orally, 1 h before induction of peritonitis with carrageenan (Cg, 500 µg/cavity) or of its vehicle (Vh, 0.9% NaCl solution, 0.1 mL/cavity). (**A**) Total number of leukocytes. (**B**,**C**) Number of neutrophils and mononuclear cells, respectively, expressed as percent of the total leukocytes (2.14 × 10^3^ ± 0.14 × 10^3^/mL). *, *p* < 0.05; **, *p* < 0.01, vs. carrageenan (One-way ANOVA followed by Bonferroni’s test). n (number of animals) = 10 for all panels. “+” means in presence of.

Evaluation of the anti-inflammatory effect of EOHc on peritonitis induced by carrageenan was performed. In order to achieve that, a dose response curve for the effect of EOHc (10, 30, 100, and 300 mg/kg) on peritoneal exudate leukocyte counts was used. The inflammatory cells were predominantly neutrophils, which contributed 68.25% (Total count: 2.14 × 10^3^ ± 0.149 × 10^3^/mL; Figure 1A.) of total cell count in peritoneal exudate (Figure 1B), whereas mononuclear cells contributed about 18% of the total leukocyte population (Figure 1C). Neutrophil count in extracellular exudate was significantly reduced by 10, 30, 100, and 300 mg/kg EOHc (*p* < 0.05; ANOVA and Bonferroni post hoc test) of the total leukocyte population by 15.00%, 43.29%, 65.52%, and 72.83%, respectively (Figure 1B).

EOHc (30–300 mg/kg) significantly inhibited leucocyte migration (Figure 1A). This inhibition was significant (*p* < 0.05, ANOVA and Bonferroni post hoc test) for doses ≥ 30 mg/kg; it had a half maximal effective concentration (EC_50_) of 36.88 mg/kg (Table 1) and reached a level near saturation at 300 mg/kg, with a decrease from 2.14 × 10^3^ ± 0.14 × 10^3^ (control) to 0.94 × 10^3^ ± 0.06 × 10^3^ leukocytes/mL.

Concerning the inhibition of several types of leukocytes, EOHc inhibited neutrophils migration with characteristics of inhibition similar to those imparted to total leukocytes (Figure 1B). This inhibition, with an EC_50_ of 24.15 mg/kg (Table 1), was significant for doses ≥10 mg/kg (*p* < 0.05, ANOVA and Bonferroni post hoc test), and reached near saturation at 300 mg/kg, at which dose the neutrophil count decreased from 68.25 ± 1.45% to 18.53 ± 1.85% of control leukocytes count (2.14 × 10^3^ ± 0.14 × 10^3^/mL). The EC_50_ for inhibition of leukocyte migration did not differ from that for neutrophil (*p* > 0.05, Student’s *t* test).

EOHc (100 and 300 mg/kg) induced numerical decrease of mononuclear and mast cells migration, which, however, did not reach statistical significance (*p* > 0.05, ANOVA and Bonferroni post hoc test).

### 2.2. Effect of EOHc on Myeloperoxidase Activity, Nitrite and Cytokines Dosage

The EOHc (10–300 mg/kg) effect on myeloperoxidase (MPO) activity and nitrite concentration at the 3rd and 5th h after inflammation induction was investigated. The temporal course (1–24 h) of the effect of EOHc (100 mg/kg) on MPO activity and nitrite concentration was also evaluated. Additionally, the effect of 100 mg/kg of EOHc on cytokines secretion at the 3rd and 5th h after inflammation induction was determined.

#### 2.2.1. Myeloperoxidase Activity and Nitrite Dosage

**Figure 2:**
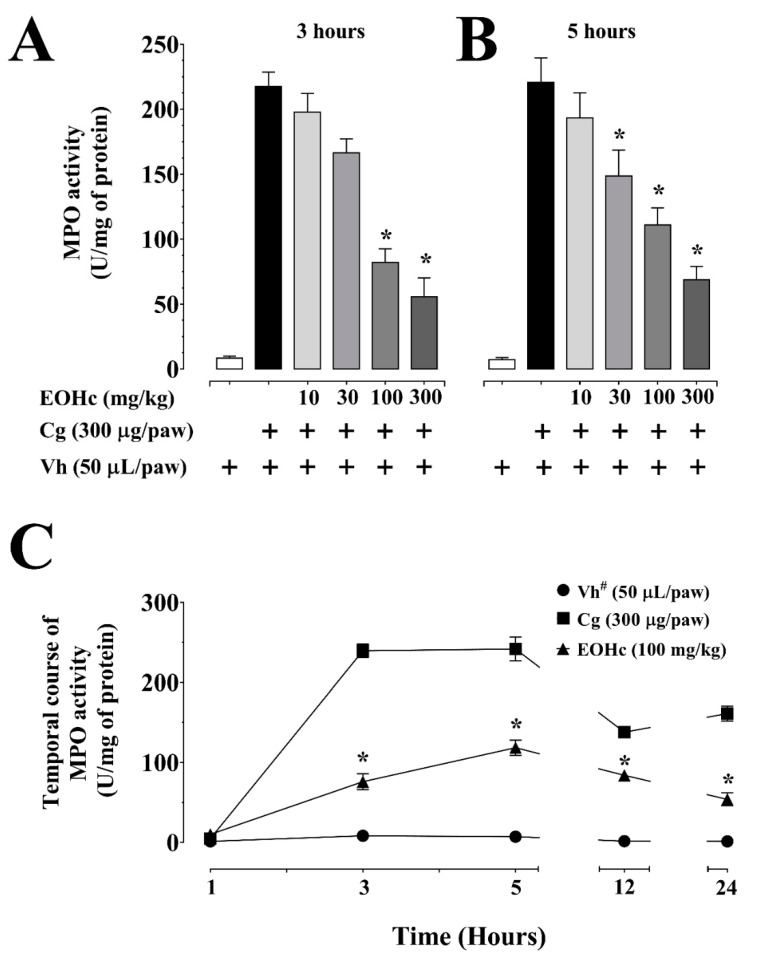
Inhibitory effects of essential oil of *Hyptis crenata* (EOHc) on myeloperoxidase (MPO) activity. EOHc or the vehicle (0.1% Tween 80) was administered, orally, 1 h before carrageenan intra-plantar injection for edema induction. Effect of EOHc (10–300 mg/kg, p. o.) at 3 h (**A**), and at 5 h (**B**) of intra-plantar injection of carrageenan (Cg, 300 µg/paw) or of its vehicle (Vh, 0.9% NaCl solution, 50 µL/paw). (**C**), effect of EOHc (100 mg/kg, p. o.) on temporal course (1–24 h) of intra-plantar injection of carrageenan. The MPO dosage was normalized by protein amount (mg). #, vehicle only. *, *p* < 0.05, vs. control, carrageenan (One-way or two-way ANOVA followed by Bonferroni’s test). n (number of animals) = 8–10 for all panels. “+” means in presence of.

EOHc showed an EC_50_ of 50.74 mg/kg (Table 1) in inhibiting MPO activity at the 3rd h after inflammation induction with carrageenan (Figure 2A). This inhibition was significant (*p* < 0.05, ANOVA and Bonferroni post hoc test) for doses of EOHc of 100 and 300 mg/kg, which inhibited carrageenan induced MPO activity (217.4 ± 11.2 U/mg of protein, n = 9) by 62.24 ± 4.87% and 74.35 ± 6.62%, respectively. 

All values of MPO activity in the presence of the carrageenan challenge, with or without EOHc, were significantly different from control MPO activity (8.46 ± 1.64 U/mg protein, n = 8), showing that the blockade of this activity was not complete. Regarding the 5th h, the inhibitory effect of EOHc was also significant for doses ≥ 30 mg/kg (Figure 2B). On the temporal course, EOHc at 100 mg/kg inhibited MPO from 3 h to 24 h (Figure 2C). At 3, 5, 12 and 24 h, EOHc inhibited 68.21 ± 4.15%, 50.93 ± 3.96%, 39.05 ± 3.67%, and 66.47 ± 5.0%, respectively, of the carrageenan-induced MPO activity. 

**Figure 3:**
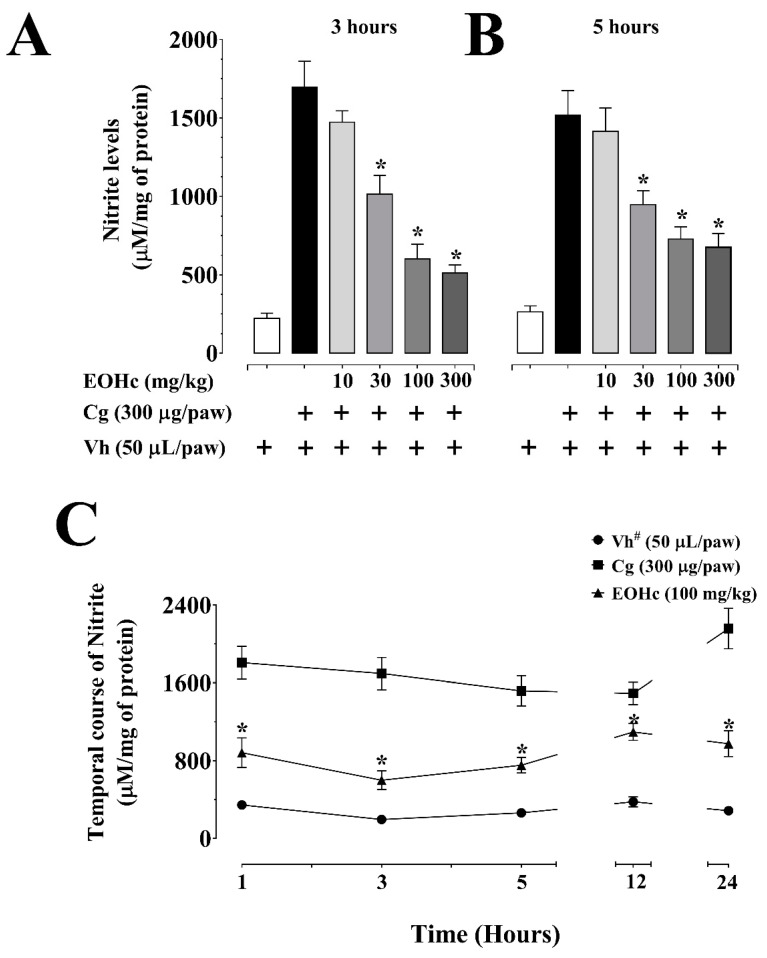
Inhibitory effect of essential oil of *Hyptis crenata* (EOHc) on nitrite levels. EOHc or the vehicle (0.1% Tween 80) was administered, orally, 1 h before carrageenan intra-plantar injection for edema induction. Effect of EOHc (10–300 mg/kg, p. o.) at 3 h (**A**), and at 5 h (**B**) of edema induced by intra-plantar injection of carrageenan (Cg, 300 µg/paw) or of its vehicle (Vh, 0.9% NaCl solution, 50 µL/paw). (**C**) effect of EOHc (100 mg/kg, p. o.) on temporal course (1–24 h) of intra-plantar injection of carrageenan. The nitrite dosage was normalized by protein amount (mg). #, vehicle only. * *p* < 0.05, vs. control, carrageenan (One-way or two-way ANOVA followed by Bonferroni’s test). n (number of animals) = 8–10 for all panels. “+” means in presence of.

In addition, EOHc (30–300 mg/kg) significantly inhibited nitrite concentration elevation (Figure 3A–C). EOHc showed EC_50_ of 29.36 mg/kg (Table 1) at the 3rd h after inflammation induction with carrageenan (Figure 3A). The inhibition was significant (*p* ≤ 0.05, ANOVA and Bonferroni post hoc test) for doses of EOHc of 30–300 mg/kg, which inhibited carrageenan-induced nitrite concentration (1696 ± 16.3 μM/mg of protein, n = 9) by 40.21 ± 7.06%, 64.64 ± 5.64%, and 69.85 ± 3.0%, respectively. This value of nitrite concentration after the carrageenan challenge in the presence of EOHc (100 mg/kg) was not significantly different from control (222.7 ± 31.84 μM/mg of protein, n = 7). Regarding the 5th h, the inhibitory effect of EOHc was significant for doses ≥ 30 mg/kg (Figure 3B). On the temporal course, EOHc at 100 mg/kg inhibited nitrite production (Figure 3C). This inhibition corresponded to 51.2 ± 8.5%, 64.64 ± 5.68%, 50.39 ± 5.32%, 26.54 ± 5.79%, and 54.79 ± 6.15% of the respective values in carrageenan-challenged animals at 1, 3, 5, 12, and 24 h after challenge, respectively. 

#### 2.2.2. Cytokines

To further investigate whether the mechanism underlying the inhibitory effects of EOHc on the development of carrageenan-induced paw edema involves modulation of inflammatory cytokine and NO production in mice, the concentration of some cytokines in the paw tissue were determined at 3rd (180 min.) and 5th h (300 min.) after carrageenan injection. 

**Figure 4:**
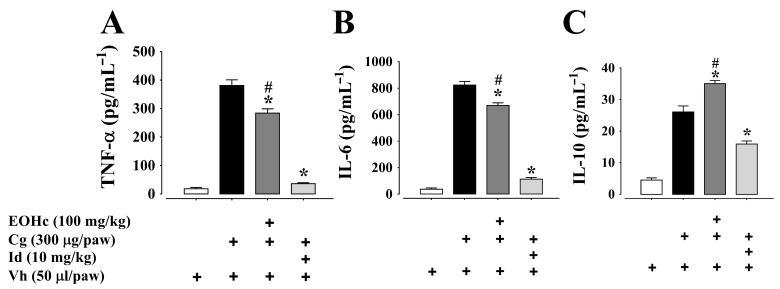
Effect of essential oil of *Hyptis crenata* (EOHc) 100 mg/kg on TNF-α (**A**), IL-6 (**B**), and IL-10 (**C**) on 3rd h of edema induced by carrageenan. EOHc or the vehicle (0.1% Tween 80) was administered, orally, 1 h before carrageenan intra-plantar injection for edema induction. The dosage of cytokines was made in paw homogenate at the peak of edema (3 h) induced by intra-plantar injection of carrageenan (Cg, 300 µg/paw,) or of its vehicle (Vh, 0.9% NaCl solution, 50 µL/paw). Id, indomethacin (positive control). *, *p* < 0.05, vs. carrageenan; #, *p* < 0.05 vs. positive control (One-way ANOVA followed by Bonferroni’s test). n (number of animals) = 6 for all panels. “+” means in presence of.

**Figure 5:**
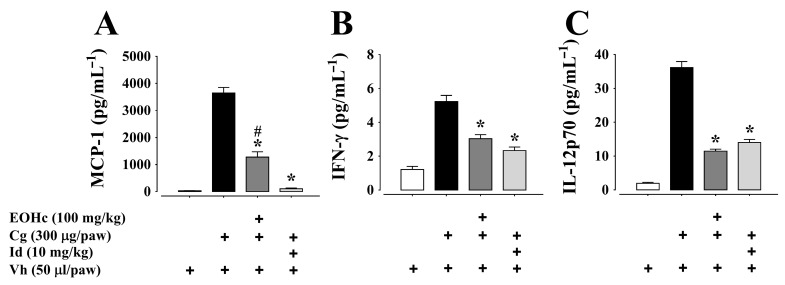
Inhibitory effect of essential oil of *Hyptis crenata* (EOHc) 100 mg/kg on MCP-1 (**A**), IFN-γ (**B**), and IL-12p70 (**C**) at 3rd h of edema induced by carrageenan. EOHc or the vehicle (0.1% Tween 80) was administered, orally, 1 h before carrageenan intra-plantar injection for edema induction. The dosage of cytokines was made in paw homogenate after 3 h of edema induced by intra-plantar injection of carrageenan (Cg, 300 µg/paw) or of its vehicle (Vh, 0.9% NaCl solution, 50 µL/paw). Id, indomethacin (positive control). *, *p* < 0.05, vs. carrageenan; #, *p* < 0.05 vs. positive control (One way ANOVA followed by Bonferroni’s test). n (number of animals) = 6 for all panels. “+” means in presence of.

Treatment with EOHc 100 mg/kg, by oral rout, markedly reduced the concentration of pro-inflammatory cytokines (TNF-α, IL-6, MCP-1, IFN-γ, and IL-12p70) and increased the concentration of IL-10 (an anti-inflammatory cytokine) in paw homogenates at the 3rd and 5th h after carrageenan administration, as compared to cytokines concentration in preparation treated only with carrageenan (*p* < 0.05; ANOVA and Bonferroni post hoc test) (Figure 4 and Figure 5). The difference of effect from the 3rd to the 5th h was not significantly different (data not shown).

Indomethacin (10 mg/kg), a drug used as a standard in anti-inflammatory evaluation, at the dose employed, had a better effect than 100 mg/kg EOHc for three out of five pro-inflammatory cytokines (IL-6, MCP-1, and TNF-α). However, for the anti-inflammatory cytokine IL-10 while indomethacin reduced its concentration, EOHc significantly (*p* < 0.05; ANOVA and Bonferroni post hoc test) increased it (Figure 4C). Concerning IFN-γ and IL-12p70, indomethacin and EOHc induced a similar decrease in these plantar cytokine concentration at both 3rd and 5th h after carrageenan administration (Figure 5B,C).

## 3. Discussion

The main finding of this study was that EOHC blunts the release of pro-inflammatory cytokines, increases the release of anti-inflammatory cytokine IL-10, and decreases tissue cellular infiltration. Additionally, it promotes a long-lasting inhibition on an increase in myeloperoxidase activity and nitrite secretion. These overall findings demonstrated the inhibition of pro-inflammation mechanisms by EOHc. The time course aspects of these effects are in accordance with the time course of antiedematogenic activity reported in our previous investigation. All these findings confirm our hypotheses that EOHc antiedematogenic activity is fully explained by its anti-inflammatory activity [10].

In the essential oil used in the present study, the most prevalent constituent was camphor (33.62%), followed by 1,8 cineole (19.76%), α-pinene (15.24%), and β-caryophyllene (8.00%). Several studies with EOHc have been published concerning the chemical constitution of this essential oil, and all samples showed similar percentage of 1,8 cineole, α-pinene, and β-caryophyllene [1,2,3,12]. In several studies, however, major differences were found in camphor amount [1,2,3,12].

Terpenic compounds are secondary metabolites occurring in aromatic and medicinal plants, and are present in large quantities in EOHc. Those compounds usually are involved with the anti-inflammatory activity of plants [13]. For instance, camphor is used in the composition of ointments (Vick^®^, Procter & Gamble, (P&G), Cincinnati, OH, USA, branch in Brazil; Salonpas^®^, Hisamitsu, Tosu, Japan, branch in Brazil) in ancillary treatment of pain, nasal congestion, and muscle inflammation [14], and its efficacy of topical analgesia has already been described [15]. Alpha-pinene has gastroprotective and antioxidant activities [8] and a potent anti-inflammatory effect by inhibiting IL-1β [16]. Alpha-pinene acts through negative regulation of mitogen-activated protein kinase (MAPKs) phosphorylation, (like ERK, extracellular signal-regulated kinase and JNK, jun N-terminal kinase), and the nuclear factor-kappa B (NF-κB) signaling pathway [17]. Additionally, alpha-pinene also inhibits overexpression of cyclooxygenase 2 (COX-2) [18]. By reducing the level of interleukin 1 beta (IL-1β) [19,20] and TNF-α, and by raising the level of IL-10, 1,8-cineole shows anti-inflammatory actions; furthermore, it has antioxidant activity [19]. In the injury of vascular endothelium induced by lipopolysaccharide (LPS), 1,8-cineole keeps the balance of pro- and anti-inflammatory cytokines, which involves the regulation of the peroxisome proliferator-activated receptor gamma (PPAR-γ) and NF-κB [21]. These previous findings partially explain the anti-inflammatory activity of EOHc [8,16,17,18,19,20,21]. Additionally, our data showed that the mixture of all these constituents in the EOHc synergistically maintained a higher anti-inflammatory activity than they show individually.

Concerning the anti-inflammatory effect, EOHc had a pharmacological potency favorable to its therapeutic use, since its EC_50_ (24.15–50.74 mg/kg, Table 1) represents only about 1.75% of its median lethal dose (LD_50_) (2000 mg/kg) [11]. Additionally, it has been documented that *Hyptis crenata* has other biological effects [22]—such as antimicrobial, fungicide [23], larvicide, and antioxidant [12]—that can be favorably associated with the anti-inflammatory effect in therapeutic uses.

Previous studies with EOHc have examined the inflammatory related pharmacological effects [2,9,10], including the antiedematogenic [10]; none of them, however, have discussed its mechanism of action. The aim of this study was to fulfill this gap and evaluate the mechanism of anti-inflammatory action of EOHc using two animal experimental models: peritonitis and paw edema. We used the peritonitis induced by carrageenan for evaluation of neutrophil migration [24], and paw edema induced by carrageenan for investigation of other components of the mechanism: MPO activity, nitrite, and cytokines quantifications [25].

In the peritoneal cavity, carrageenan induces peritonitis with great neutrophil migration and intraabdominal liquid extravasation and accumulation. EOHc markedly decreased the leucocyte migration to peritoneal cavity. Among leucocytes, neutrophils are the first cells to migrate to the injured tissue, attracted by chemotactic factors that help in recruiting them to the inflammation site [26,27]. They have specific membrane receptors that recognize microorganisms and dead cells, making them a target for phagocytosis [28,29]. Neutrophils infiltration at the site of inflammation however may aggravate the inflammatory process by producing excessive amounts of proteolytic enzymes, reactive oxygen species, eicosanoids, and cytokines [30,31]. It is known that neutrophils release molecules, such as lysosomal enzymes known as MPO to destroy the offending agent [32].

MPO, an important mechanism in inflammation, is also a marker of acute inflammatory processes [33]. Accordingly, the fact that in the present study, EOHc significantly decreased MPO activity in carrageenan-injected tissue shows the unequivocal anti-inflammatory activity of this essential oil. Since measurements of tissue levels of MPO can indirectly indicate neutrophil recruitment, the effective reduction in MPO activity by EOHc indicates that EOHc inhibits the migration of neutrophils into the inflamed tissue [34,35,36].

Macrophages also help in process of inflammatory defense. These cells release substances such as NO into the inflammation site, which, even having an important participation in the defensive aspects of inflammation, when reacting with peroxides, however, results in high destructive potential [37,38]. Inducible nitric oxide synthase (iNOS), synthesized by inflammatory cells, especially macrophages, greatly increases NO in inflamed tissues [39], leading to vasodilation and tissue damage [40,41,42]. NO was indirectly quantified through nitrite dosage [43]; this quantification showed that EOHc promoted a decrease in NO release, thus demonstrating the anti-inflammatory activity of this essential oil on this arm of inflammatory process. Since EOHc inhibited neutrophilic migration and decreased nitrite content from the paw (supposedly from macrophages [39]), these facts may suggest that the effect of EOHc does not involve only one cell type, but multiple anti-inflammatory mechanisms. 

The literature reports that the recruitment of leukocytes to inflammatory sites is a process largely influenced by small polypeptides known as cytokines [44]. There are two main groups of cytokines, namely, pro-inflammatory and anti-inflammatory [45]. In general, pro-inflammatory cytokines are synthesized, predominantly, by active macrophages [46], lymphocytes, and also endothelial cells and fibroblasts [47], while the anti-inflammatory cytokines are synthetized by T cells. The inflammatory process upregulates the production and secretion of pro-inflammatory (TNF-α, MCP-1, IFN-γ, IL-12p70, and IL-6) cytokines and down-regulates anti-inflammatory cytokines (IL-10) [48]. Likewise, the EOHc administration blunted the elevation of pro-inflammatory cytokines (TNF-α, MCP-1, IFN-γ, IL-12p70, and IL-6) and upregulated IL-10, an anti-inflammatory cytokine. It is noteworthy that EOHc increased the concentration of IL-10, an anti-inflammatory cytokine, while indomethacin did not.

## 4. Materials and Methods

### 4.1. Obtaining Essential Oil of Hyptis Crenata

The EOHc here employed is from the same source as in Coelho-de-Souza [10]. The plants were collected in the city of São Raimundo das Mangabeiras and identified by Dr. Oriel Herera Bonilla (Ecology Laboratory, State University of Ceara, Fortaleza, CE, Brazil). They underwent the same procedures for plant identification, oil extraction, and chemical identification as in our previous publication [9,10]. Briefly, the aerial parts (leaves and branches) were dried freely and subsequently were processed in a semi-industrial essential oil extractor (model MA 480, Marconi equipment, Piracicaba, São Paulo, Brazil) using a water steam distillation method. In the condenser that was attached to the extractor, a mixture of essential oil and water was collected, and the oil phase was separated from the water phase. The oil phase, essential oil, was stored in a refrigerator (approximately 4 °C) in amber glasses until use. The yield of EOHc was 1.47% of the weight of the dry part of the plant. The analysis of chemical constituents was done at the Technological Development Park of the Federal University of Ceara, Fortaleza, Ceara, Brazil using gas chromatography/mass spectrometry (GC/MS). The chromatographic analysis was performed on a Hewlett-Packard 6971 (Palo alto, CA, USA) under the following analytical conditions: the column used was a dimethylpolysiloxane DB-1 fused silica capillary column (30 m × 0.25 mm; 0.1 µm); carrier gas: helium (1 mL/min); injector temperature: 250 °C; detector temperature: 200 °C; column temperature: 35–180 °C at 4 °C/min and then 180–250 °C at 10 °C/min; and mass spectra: electronic impact 70 eV. These compounds were identified using a mass spectral library search.

### 4.2. Drugs and Solutions

All salts and drugs used for this study were of analytical purity purchased from Sigma-Aldrich (Darmstadt, Germany), Merck (Darmstadt, Germany), Reagem (Porto Alegre, RS, Brazil), or Vetec (Fortaleza, CE, Brazil). A Mouse Inflammation-Kit (BD Cytometric Bead Array-CBA) was used for detection and labeling of inflammatory cytokines: interleukin 6 (IL-6), interleukin 10 (IL-10), monocyte chemotactic protein 1 (MCP-1), interferon gamma (IFN-γ), tumor necrosis factor alpha (TNF-α), and interleukin 12p70 (IL-12p70) were from Becton, Dickinson, and Company BD Biosciences (Juiz de Fora, MG, Brazil).

### 4.3. Animals and Treatment

This work used male germfree mice of the *Swiss* strain acquired from Christus University. The animals were housed at the State University of Ceara (UECE) at a controlled temperature of 22 ± 3 °C, in a box of polypropylene with free access to water and food until their use. All the experimental protocols were approved by the Committee on Ethics in the Use of Animals of the UECE (protocol number 2960651/2015).

The EOHc was prepared in sterile saline containing Tween 80 (0.1%, *v*/*v*) followed by automatic stirring. Immediately after stirring, the solution was administered orally.

### 4.4. Peritonitis Induction

After 1 h of administration of EOHc, peritonitis induction was performed, for total and differential leucocytes count, by intraperitoneal injection of carrageenan (500 μg) diluted in 0.1 mL of sterile saline. The animals were sacrificed 4 h posteriorly to the induction, and the peritoneal cavity was open and washed with 3 mL of saline. The peritoneum was massaged, and the exudate was collected, in sterile conditions, for total and differential leucocytes counting by optical microscopy. For total leucocytes counting, 20 μL of the peritoneal fluid was diluted in 380 μL of Turk’s reagent and put in a Neubauer chamber under optical microscope with objective of 40×.

The differential leukocyte count was also performed under light microscopy (Eclipse E 200, Nikon). For this purpose, cells were prepared by cytocentrifugation (Cytocentrifuge. Cytopro 7620, Wescor, UT, USA) of the intraperitoneal fluid at 1000 RPM for 10 min followed by Fast Panotic LB staining.

### 4.5. Myeloperoxidase Activity

For the evaluation of MPO activity, a dose response curve (dose range: 10–300 mg/kg of corporal weight) was made. In order to determine the most appropriate moment for the dose-response curve, the time course of the effect of 100 mg/kg EOHc on MPO was observed. It included the EOHc effect on MPO activity at the following time intervals: 1st, 3rd, 5th, 12th, and 24th h. MPO activity of neutrophils was evaluated following a protocol previously described [10]. Briefly, EOHc was administered 1 h before edema induction. After 1 h, 3 h, 5 h, 12 h, or 24 h of carrageenan-induced edema (300 µg/paw, intra-plantar injection), the subplantar tissue was removed and immediately stored at −80 °C for posterior dosage of MPO. At the time of dosage, tissue samples were thawed and incubated in 0.5% CTAB solution (cetyltrimethylammonium bromide, 50 mg of tissue/mL), homogenized, and centrifuged at 10,000× *g*, 4 °C, for 7 min. The supernatant was transferred to an Eppendorf tube and submitted to heat shock in three freeze-thaw stages (−20 °C, 10 min. each), homogenized again, and centrifuged (High Speed Refrigerated Centrifuge, Model TGL-20MB, Daiki Ltd., Hunan, China) for 7 min at 4 °C (10,000× *g*). Finally, 30 µL of supernatant was mixed with 200 µL of reading solution (0.167 mg/mL of dihydrochloride of o-dianisidine, 0.2 mM of potassium phosphate buffer, and 0.0006% hydrogen peroxide (H_2_O_2_)). The absorbance of this mix was measured in a spectrophotometer (Multimode microplate reader, model VICTOR Nivo, PerkinElmer, Massachusetts, EUA) at 450 nm for MPO activity based on the optical density that changes due to the decomposition of H_2_O_2_ in the presence of o-dianisidine. The results were expressed as an MPO activity unit. One unit of MPO activity was defined as the amount of enzyme that degrades 1.0 μmol of H_2_O_2_ per min. at 25 °C, expressed as MPO × 10^3^/mg protein.

### 4.6. Concentration of Nitrite (Griess Reaction)

Nitric oxide (NO) production was measured from the subplantar homogenate. For this protocol, a dose response curve was made for doses of 10, 30, 100, and 300 mg/kg of body weight, orally. EOHc was administered 1 h before edema induction. Subplantar tissue was used to assess nitric oxide production. The time course of the effect of 100 mg/kg EOHc on NO production was observed, which included measurements at the 1st, 3rd, 5th, 12th, and 24th h using the colorimetric method of Griess [49]. The absorbance of Griess’s reagent and sample (1:1 ratio) was measured at 540 nm using a Biotech Synergy HT microplate reader. Protein dosage was determined by the Lowry method [50] and the results of NO production were expressed as μM/mg of protein. Nitrite concentration was calculated using standard solutions of sodium nitrite (NaNO_2_).

### 4.7. Cytokines Quantification 

EOHc was administered 1 h before edema induction. The quantification of cytokines was performed on the 3rd and 5th h after carrageenan edema induction from the mixture of the subplantar homogenate with the Mouse Inflammation Kit (BD-CBA) according to manufacturer’s recommendations. Briefly, the samples were incubated with a protease inhibitor (1 μL per 400 μL of sample) for 1 h at 4 °C. Then, the samples were sonicated for 7 min and centrifuged at 14,000 RPM for 15 min at 4 °C. The supernatant was read on a flow cytometer calibrated with the D Bead Setup solution for investigation of cytokines. After acquiring the samples on the flow cytometer, the results were generated using FCAP Array^TM^ software and expressed in pg/mL^−1^.

### 4.8. Statistical Analysis

Data are reported as mean ± standard error of the mean (SEM). The results that present probability of occurrence of a nullity hypothesis less than 5% were considered statistically significantly different. The significance (*p* ≤ 0.05) of the results was assessed by a one-way or two-way analysis of variance (ANOVA), followed by Bonferroni’s post hoc test.

## 5. Conclusions

In conclusion, the present study demonstrated that EOHc has an anti-inflammatory effect due to inhibitory action on several branches of the cascade of the inflammatory mechanism. It not only demonstrates that EOHc has, with a high efficacy, a long-lasting antiedematogenic and anti-inflammatory effect, but, due to its low toxicity, as far as results obtained in mice could be translated to humans, suggests this agent as a likely potential candidate for an anti-inflammatory medicine.

## Figures and Tables

**Table 1:** EC_50_ values of essential oil of *Hyptis crenata* on mediators of inflammation. (n^1^ = 8–10).

Experimental Model	Test	EC_50_ (mg/kg)
Peritonitis induced by carrageenan	Leucocytes migration	36.88
Peritonitis induced by carrageenan	Neutrophilic migration	24.15
Paw edema by carrageenan	MPO activity	50.74
Paw edema by carrageenan	Nitrite levels	29.36

n^1^, number of animals.

## Data Availability

Not applicable.
